# Association of Access to Crisis Intervention Teams With County Sociodemographic Characteristics and State Medicaid Policies and Its Implications for a New Mental Health Crisis Lifeline

**DOI:** 10.1001/jamanetworkopen.2022.24803

**Published:** 2022-07-15

**Authors:** Helen Newton, Tamara Beetham, Susan H. Busch

**Affiliations:** 1 Yale School of Public Health, New Haven, Connecticut

## Abstract

**Importance:**

The mental health crisis lifeline 988 will begin operating July 16, 2022. In the absence of appropriately trained first responders, including crisis intervention teams (CITs), persons experiencing behavioral health crises face the risk of incarceration and even death.

**Objective:**

To assess county-level access to CIT in 2015 and 2020 and its association with area characteristics and state policies in 2020.

**Design, Setting, and Participants:**

This cross-sectional study included 10 430 facilities from the 2015 National Directory of Mental Health Treatment Facilities and 10 591 facilities from the 2020 National Directory of Mental Health Treatment Facilities, attributed to 3142 US counties.

**Exposures:**

Area measures included need (suicide, drug-related overdose mortality), rurality, and demographic characteristics. State-level policies included 5 Medicaid policies enacted prior to 2020 and 2 recent policies intended to assist implementation of the 988 lifeline.

**Main Outcomes and Measures:**

Whether there was at least 1 facility that reported offering a CIT that handled acute mental health issues at the facility or off-site for each county in 2015 and, separately, in 2020.

**Results:**

Most US residents (88%) resided in a county with at least 1 facility offering CIT, although half of US counties had no facility offering CIT (2015: 1537 of 3142 [49%]; 2020: 1512 [48%]). Almost 1 in 5 counties, representing 9% of the population, experienced a change in county-level access from 2015 to 2020. Unadjusted analyses indicated residents of counties without vs with CIT access were more likely to be older and uninsured (top quartile of percentage of residents aged >55 years: 502 of 1512 [33%] vs 283 of 1630 [17%]; *P* < .001; top quartile of percentage of residents uninsured: 500 [33%] vs 285 [17%]; *P* < .001) and were more likely be rural (frontier: 500 [33%] vs 144 [9%]; *P* < .001). Similar results, excluding counties in the top quartile of residents aged older than 55 years, were found in adjusted analyses. Counties without vs with CIT access were less likely to be in states that expanded Medicaid (788 [52%] vs 1102 [68%]; *P* = .01) and in states that allow Medicaid to pay for short-term stays in psychiatric hospitals (34 [2%] vs 73 [4%]; *P* = .02). Other Medicaid-related associations were not statistically significant in adjusted analyses.

**Conclusions and Relevance:**

In this study, most US residents lived in counties with access to at least 1 CIT, but fewer than half of US counties had such access. Policies to encourage facilities in rural counties to offer CIT may help realize the potential of the new lifeline.

## Key Points

**Question:**

How did county-level access to crisis intervention team (CIT) services change in 2020 compared with 2015, and was CIT access associated with area and state policy characteristics in 2020?

**Findings:**

In this study of more than 10 000 facilities in 3142 US counties, most US residents lived in counties with access to at least 1 mental health facility offering CIT services; however, fewer than half of US counties had such access. Counties without CIT access tended to have populations that were older and more likely to be uninsured, and they were more likely to be rural.

**Meaning:**

Encouraging facilities in rural counties to offer CIT may help realize the potential of policies to address behavioral health crises, such as the new mental health crisis lifeline.

## Teaser

This cross-sectional study assesses county-level access to crisis intervention teams (CITs) in 2015 and 2020 and its association with area characteristics and state Medicaid policies.

## Introduction

Individuals experiencing a behavioral health crisis are at risk for self-harm and can sometimes require evaluation and intervention, but they are also at risk for incarceration and mortality when first responders lack adequate training.^1^ While crisis services were a core component of the community behavioral health system established by the 1963 Community Mental Health Act, these programs have not been available in many communities.^2,3^ Over the past 5 years, access to effective crisis care has become more urgent as rates of drug-related overdose, suicidal ideation, and other behavioral health crises have increased.^4,5^ In addition, there is growing recognition that individuals with mental health or substance use disorders are at higher risk of arrest.^6^

Crisis intervention teams (CITs) are 1 of 3 core components of an effective behavioral health crisis care system. In addition to a mobile crisis intervention team, Substance Abuse and Mental Health Services Administration (SAMHSA) guidelines require a call center open 24 hours a day and 7 days a week as well as crisis stabilization services.^7^ Crisis services are currently initiated by calling 911 or another crisis line, such as a suicide lifeline. While most calls (approximately 80%) can be managed by phone, 10% to 20% require in-person response.^8^ In this case, CITs are sent to assess the client and provide transport to stabilization services, if necessary. Approximately 1 in 3 in-person visits from the CIT require stabilization services, which can include hospitalization. Encounters handled by untrained law enforcement are more likely to result in arrest than referral to treatment.^9^

Although the CIT model originated as a partnership between a local police department and mental health center, CIT and other crisis services are increasingly housed in specialty mental health facilities.^10^ In 2019, approximately 15% of police departments reported having a CIT program, while 52% of mental health treatment facilities reported offering a CIT service.^11,12^ In part, this is because the goals of CIT and crisis care have changed to focus on treatment access and engagement rather than solely public safety. CIT is now considered an important “front door” for subsequent behavioral health treatment, and crisis teams will follow-up with callers and schedule appointments to ensure treatment engagement and retention. Colocating crisis services with other specialty behavioral health services facilitates treatment after stabilization. As with many other specialty mental health services, CITs are often grant funded. Crisis intervention services are reimbursable by Medicaid in most states, although licensure of personnel qualified to provide reimbursable service varies.

US Congress recently authorized a new mental health crisis lifeline, 988, to go live July 16, 2022. This new crisis line replaces the national suicide lifeline and expands the scope of services that callers can have addressed, including substance use issues and other behavioral health crises. The new crisis lifeline is the latest effort in a series of federal and state efforts to improve access to effective crisis care. Past efforts include the recent Certified Community Behavioral Health Clinic (CCBHC) demonstration and expansion programs, which required participating behavioral health clinics to provide crisis management in addition to other services.^13^ More recently, the American Rescue Plan (ARP) authorized the Centers for Medicare & Medicaid Services (CMS) to make funding available for states to plan their implementation and administration of mobile crisis services.^14^ In this study, we examined access to 1 crisis service likely to affect 988 implementation: CITs.

## Methods

### Study Design

This study is a cross-sectional analysis of responses to the National Mental Health Services Survey (N-MHSS) reported in the 2015 and 2020 National Directory of Mental Health Treatment Facilities (hereafter, the directory). We considered 1 measure of county-level access to CITs by measuring whether at least 1 mental health facility offered a CIT in each US county. This measure likely estimates an upper bound of access, as some CITs may only serve select areas within a county or may not have personnel or other resources to serve all-county need. We then examined unadjusted and adjusted associations between access to CIT with area and state Medicaid policy characteristics. This study followed the Strengthening the Reporting of Observational Studies in Epidemiology (STROBE) reporting guidelines. The Yale institutional review board considered this study non–human participant research and therefore waived review and approval.

### Participants

Study participants included all facilities that responded to SAMHSA’s N-MHSS and chose to be included in the directory. The N-MHSS is an annual survey fielded April through June that collects administrative data from all known public and private mental health treatment facilities nationally, excluding those operated by Department of Defense, jails and prisons, and independent practitioners.^15^ Select survey responses are published in the directory along with facility name and street address. Responding facilities can choose to not be included in the directory (approximately 20%).

### Main Outcomes

For each US county in the 50 states and District of Columbia, we measured whether there was at least 1 mental health treatment facility offering a CIT. We considered responses to the following question included in the N-MHSS: “Does this facility offer a crisis intervention team that handles acute mental health issues at this facility and/or off-site?”^11^ We then used facility street address included in directory to determine whether there was at least 1 facility that answered yes in 2015 and, separately, in 2020 for each county. The response rate for the relevant question was 99.4% in 2020.^16^

### Area Characteristics and State Medicaid Policies

For each county, we constructed binary variables indicating whether the county was in the top quartile of counties in terms of the percentage of residents older than 55 years, uninsured, and unemployed. We also considered whether the county was in the top quartile of counties for 6 racial and ethnic categories (share of residents who are American Indian or Alaska Native, Asian, Black, Hispanic, Native Hawaiian or Pacific Islander, and White). We included data on county-level racial and ethnic characteristics because minoritized racial and ethnic communities are especially at risk for police violence and typically experience greater barriers to accessing mental health care relative to White communities.^17^ We also constructed a binary variable indicating whether the county was in the top quartile of a county-level neighborhood residential segregation index. Residential segregation indices measure the evenness with which 2 groups (racial or ethnic minority group and White) are distributed across the census tracts that make up a county; our indicator identifies those counties with the most segregation.^18^ We also considered need for services, such as whether the county was in the top quartile of county-level suicide and drug-related overdose mortality per 100 000 population rates, and county rurality (measured using Rural-Urban Commuting Area [RUCA] codes: metropolitan [urban], RUCA codes 1-3; micropolitan [rural], RUCA codes 4-7; and frontier [rural], RUCA codes 8-10). All area measures were constructed using publicly available data from the 2020 to 2021 Area Health Resource File and County Health Rankings 2020.^19,20^ Although most variables had complete information for each county, County Health Rankings suppress values for select counties because of measure unreliability: 351 counties had missing residential segregation index values, 763 counties had missing suicide mortality rates, and 1420 counties had missing drug-related overdose rates.

We considered 5 state-level Medicaid policies enacted before 2020 to assess whether they were associated with CIT access. We included policies that could either encourage demand for crisis intervention services (whether the state had expanded Medicaid by 2020) or increase supply of crisis intervention services through federally supported demonstration programs or grant funding and other payments.

We examined 2 demonstration programs awarded to states: Medicaid Section 1115 waivers, which give states flexibility to develop their Medicaid programs to better serve their Medicaid enrollees, and the CCBHC demonstration planning grants, which awarded grants to 22 states in 2016. We only considered Section 1115 waivers that focused on behavioral health, were awarded before 2020, and were active during the study period.^21^ We examined associations between presence of any behavioral health Section 1115 waiver and also subcategories of Section 1115 waivers: those that focus only on waiving Medicaid payments to institutes of mental disease (IMD) for substance use disorder or mental health treatment, those that focused on expanding eligibility for Medicaid behavioral health services, those focused on reforming the behavioral health delivery system, and those that expanded community-based benefits. The CCBHC was a novel Medicaid demonstration that required designated clinics to provide a comprehensive range of mental health and substance use treatment services, including crisis management, starting in 2017.^13^ In 2016, 22 states were given 1-year planning grants to develop their specialty behavioral health systems in preparation for the 2017 demonstration program, which was awarded to 8 states.

Because a state could also expand crisis intervention supply via federal grants or disproportionate share hospital (DSH) payments to psychiatric hospitals, we also included information on state DSH payments and identified state receipt of SAMHSA grant awards for suicide prevention, crisis intervention, or diversion from fiscal years 2015 to 2020 using the Tracking Accountability in Government Grants system.^22,23^

We also considered 2 recent policies intended to assist 988 implementation to assess whether support matched need: state receipt of 2021 ARP CIT development grants and the status of recent state legislation to fund 988.^14,24^ The ARP development grants, awarded by CMS, provided planning resources to 20 state Medicaid agencies to assess need for and develop crisis intervention programs. State legislation to finance 988 refers to any legislation that states have passed, or is pending, that could finance implementation and administration of 988 lifelines through phone bill taxes (similar to current financing mechanisms for 911 services) or other state funds.

### Statistical Analysis

We measured unadjusted associations between area and state Medicaid policy characteristics and 2020 county-level CIT services using Wald χ^2^ tests that adjusted SEs to account for correlation within states. Tests for significance were 2-tailed, and *P* ≤ .05 was considered statistically significant. We used logistic regression models to compute the adjusted associations between area and state Medicaid policy characteristics and adjusted SEs to account for correlation within states (results from logistic regression models available in eTable 2 in the Supplement). We excluded measures of suicide and drug-related overdose mortality from regression models because the number of counties with missing observations was greater than 20% in both cases. Sensitivity analyses using the subsample of counties with complete information for suicide and drug-related overdose are available in eTable 3 in the Supplement. Our main specification included 2790 counties with complete information for remaining covariates. For ease of interpretation, we transformed logistic model coefficients into percentage point change in estimated probability. All analyses were completed using Stata version 15.1 IC (StataCorp).

## Results

### Changes in County Access to Crisis Intervention Team Services in 2015 and 2020

**Figure 1.**
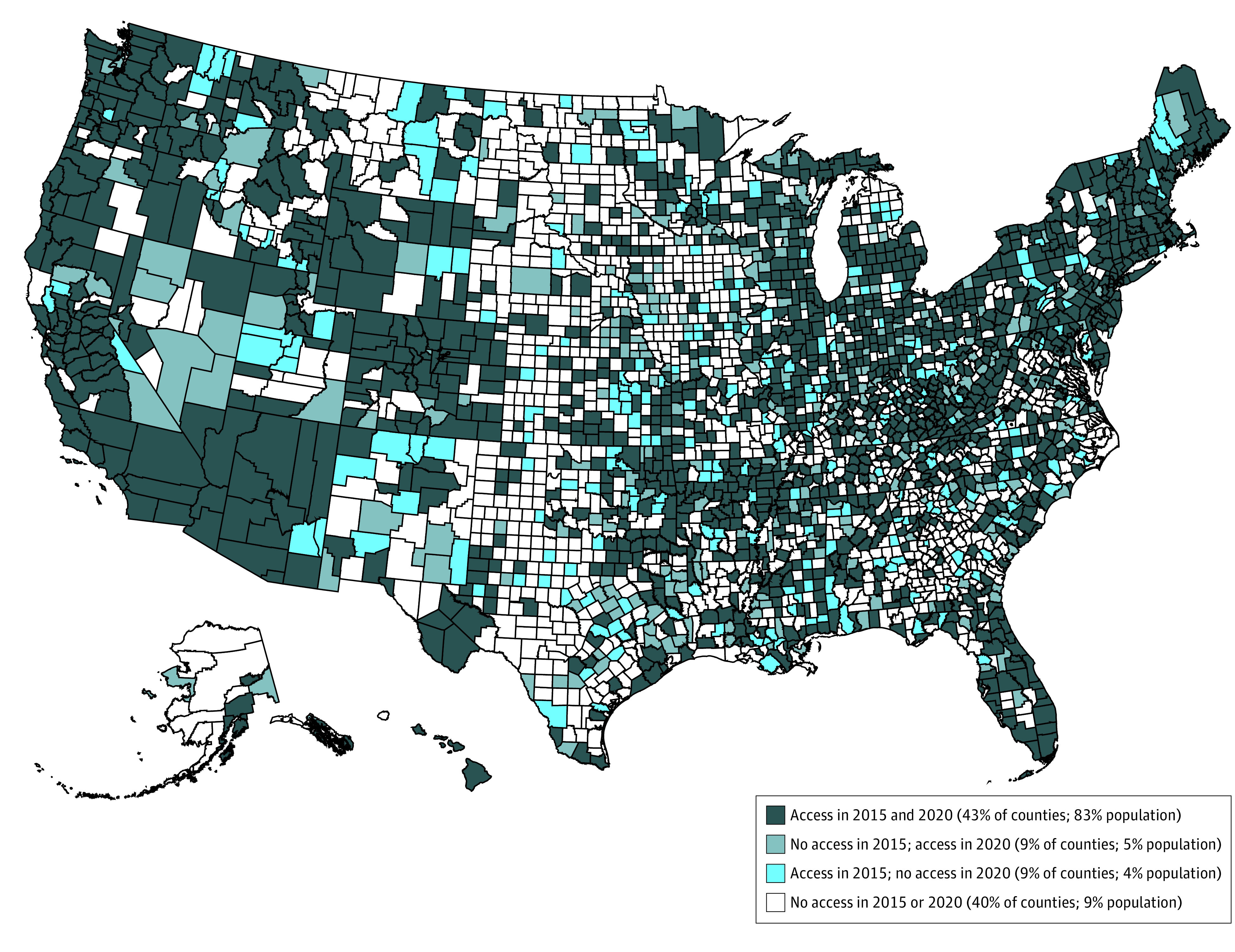
Changes in County-Level Access to Crisis Intervention Teams (CITs) The 2015 and 2020 National Directory of Mental Health Treatment Facilities included information from 10 430 and 10 591 facilities, respectively. This figure uses responses from the 5050 and 5238 facilities listed in 2015 and 2020 National Mental Health Treatment Directories who responded yes to the question “Does this facility offer a crisis intervention team that handles acute mental health issues at this facility and/or off-site?” For each US county, we determined whether there was at least 1 facility that answered yes to this question. Half of counties had no facility offering CIT: 1537 of 3141 (49%) in 2015 and 1512 (48%) in 2020. Data from 2015 and 2020 was used to categorize counties into 1 of 4 categories: had no access to CIT in either year, lost access, gained access, and had access in both years. The share of the 2020 population that resided in each category of counties was then calculated.

Directories included information for 10 430 and 10 591 facilities in 2015 and 2020, respectively, representing 72% and 75% of all facilities that provided mental health treatment services in the 50 US states and DC in 2015 and 2020.^25^ While most of the population (88%) had access to crisis intervention team services, half of US counties had no facility offering CIT services: in 2015, 1537 of 3142 counties (49%) and, in 2020, 1512 counties (48%) did not have any CIT access. Approximately 1 in 5 counties (representing 9% of the population) experienced a change in access in 2020 compared with 2015, although the net effect did not change, with a similar share of counties losing and gaining access to CIT during this period (Figure 1 and eTable 1 in the Supplement).

### Area Characteristics Associated With Access to CIT Services in 2020

In unadjusted analyses, counties without CIT access in 2020 had high need compared with those with access: 282 of 1512 counties (31%) without access were in the top quartile of county suicide mortality rate compared with 312 counties (21%) with CIT access (*P* < .001) (Table). Counties without vs with access to CIT had residents who were more likely to be older and uninsured and were also more likely be rural (top quartile of percentage of residents aged >55 years: 502 [33%] vs 283 [17%]; *P* < .001; top quartile of percentage of residents without insurance: 500 [33%] vs 285 [17%]; *P* < .001; frontier: 500 [33%] vs 144 [9%]; *P* < .001). Counties without vs with CIT access tended to be less racially diverse with respect to Asian and Native Hawaiian or Pacific Islander residents (top quartile of percentage of Asian residents: 182 [12%] vs 603 [37%]; *P* < .001; top quartile of percentage of Native Hawaiian or Pacific Islander residents: 318 [21%] vs 467 [29%]; *P* = .01). Counties without vs with access to CIT were more likely to have high levels of residential segregation (most segregated: 237 [19%] vs 460 [29%]; *P* = .002).

**Table.** County Characteristics and State Policies of Counties With and Without Access to CIT Services, 2020

Characteristic^a^	Counties with access to CIT, No. (%) (N = 3142)		*P* value^b^
	No	Yes	
Counties	1512 (48)	1630 (52)	NA
County population in 2020	40 534 438 (12)	288 949 685 (88)	NA
Top quartile of residents >55 y	502 (33)	283 (17)	<.001
Top quartile of demographic characteristics			
Uninsured	500 (33)	285 (17)	<.001
Unemployed	348 (23)	437 (27)	.09
Median household income	413 (27)	372 (23)	.17
Top quartile of race and ethnicity			
American Indian or Alaskan Native	402 (27)	383 (23)	.36
Asian	182 (12)	603 (37)	<.001
Black	362 (24)	423 (26)	.64
Hispanic	338 (22)	447 (27)	.41
Native Hawaiian or Pacific Islander	318 (21)	467 (29)	.01
White	429 (28)	356 (22)	.11
Top quartile of residential segregation	237 (19)	460 (29)	.002
Top quartile of behavioral health mortality in 2020			
Drug overdose deaths per 100 000 population	134 (26)	296 (25)	.72
Suicide deaths per 100 000 population	282 (31)	312 (21)	<.001
Rurality			
Metropolitan (urban)	384 (25)	782 (48)	<.001
Micropolitan (rural)	628 (42)	704 (43)	.61
Frontier (rural)	500 (33)	144 (9)	<.001
State Medicaid policies enacted before 2020			
Expanded Medicaid	788 (52)	1102 (68)	.01
Any Behavioral Health Section 1115 waiver	720 (48)	968 (59)	.05
IMD payment exclusion			
Substance use disorder	646 (43)	837 (51)	.15
Mental health	34 (2)	73 (4)	.02
Eligibility expansions	61 (4)	131 (8)	.19
Delivery system reforms	64 (4)	76 (5)	.83
Community-based benefit expansions	350 (23)	491 (30)	.15
2015 Certified Community Behavioral Health Clinic Demonstration Planning Grant State	720 (48)	855 (52)	.47
Top quartile of per-capita SAMHSA funding related to suicide prevention, crisis intervention, or diversion	335 (22)	405 (25)	.58
Top quartile of IMD DSH payments	369 (24)	372 (23)	.79
State Medicaid policies enacted after 2020			
American Rescue Plan CIT development planning grant state	420 (28)	617 (38)	.06
988 legislation passed or pending	517 (34)	684 (42)	.27

Abbreviations: CIT, crisis intervention team; DSH, disproportionate share hospital; IMD, Institutions of Mental Disease.

^a^ Three area characteristics have missing observations (measures of drug-related overdose deaths, suicide mortality, and residential segregation); percentages reported in this table reflect the percentage among observations with nonmissing values (1420, 763, and 351 counties with missing values, respectively).

^b^ *P* values are from Wald χ^2^ tests that adjusted SEs to account for correlation of SEs by state.

**Figure 2.**
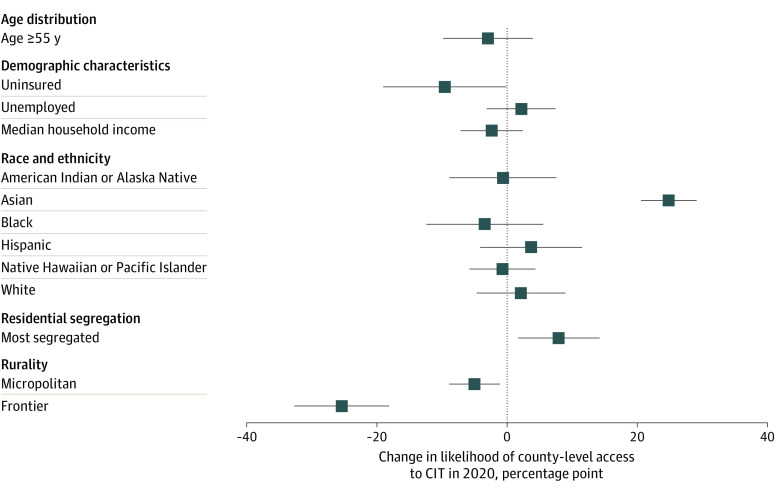
Adjusted Associations Between Area Characteristics and County-Level Access to Crisis Intervention Teams (CITs) in 2020 This figure shows the change in estimated probability and 95% CIs from a logistic regression model estimating county-level CIT in 2020 using all covariates listed in the Table, excluding measures of need. Logistic regression models were used to compute the adjusted associations between area and state Medicaid policy characteristics and adjusted SEs to account for correlation within state. All measures except rurality are binary and represent counties in the top quartile of that measure.

After adjusting for multiple area and state Medicaid policy characteristics, several factors remained statistically significantly associated with CIT access. Counties within the top quartile of percentage of residents without insurance were associated with a 10-percentage point (95% CI, 0.1- to 19-percentage point) lower likelihood of access to CIT services compared with those with lower rates of uninsurance and micropolitan and frontier rural counties were 5 (95% CI, 1 to 9) percentage points and 25 (95% CI, 18 to 32) percentage points less likely than metropolitan counties to have access to CIT services (Figure 2). Counties with high Asian populations and high levels of residential segregation were more likely to have access to CIT services.

### State Medicaid Policies Associated With Access to CIT Services in 2020

**Figure 3.**
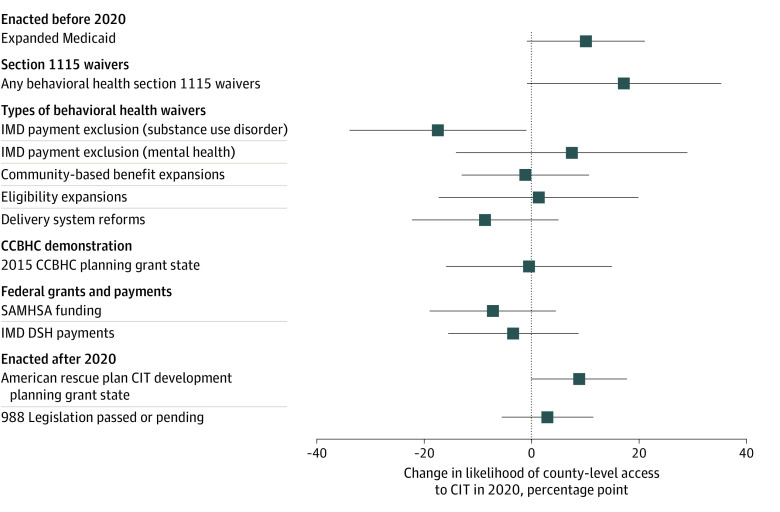
Adjusted Associations Between State Medicaid Policies and County-Level Access to Crisis Intervention Teams (CITs) in 2020 This figure shows the change in estimated probability and CIs from a logistic regression model estimating county-level CIT access in 2020 using all covariates listed in the Table, excluding measures of need. Logistic regression models were used to compute the adjusted associations between area and state Medicaid policy characteristics and adjusted SEs to account for correlation within state. All measures are binary; federal grants and payments categories represent the counties in the top quartile of that category. CCBHC indicates Certified Community Behavioral Health Clinic; DSH, disproportionate share hospital; IMD, institutes of mental disease; and SAMHSA, Substance Abuse and Mental Health Services Administration.

In unadjusted analyses, counties without vs with access were less likely to be in states that expanded Medicaid prior to 2020 (788 [52%] vs 1102 [68%]; *P* = .01) (Table) and less likely to be in states that allowed Medicaid to pay for short-term stays in psychiatric hospitals (IMD payment exclusion for mental health: 34 [2%] vs 73 [4%]; *P* = .02). We did not find a significant association between access to CIT in 2020 and receipt of 2021 ARP development grants. After adjusting for area characteristics and presence of other state Medicaid policies, we found no statistically significant associations between state Medicaid policies and access to CIT services, although direction and magnitude of the unadjusted associations remained similar (Figure 3).

## Discussion

In this national cross-sectional study, we found that half of US counties—representing approximately 1 in 9 people—did not have access to at least 1 CIT in 2020, and rural communities were the least likely to have access. We found that there was little change in the percentage of facilities reporting offering services from 2015 to 2020. Although we found the net number of counties with access did not change, approximately 9% of counties lost access and, separately, 9% gained access in 2020. This suggests there may be opportunities to increase public funding or technical support in centers in danger of losing CIT access to prevent these closures. While this study only looks at one component of crisis care, this analysis provides a lens through which to consider the current distribution of crisis services and resources to assist in the upcoming 988 implementation. We might expect fewer individuals to have effective access if within a county individuals reside far from the CIT or if some CITs within a county lack the capacity to serve the entire county, as is likely in many urban areas.

As with other mental health treatment services, access to CITs remains inequitable: counties with more uninsured residents, more residents aged 55 years or older, and the highest suicide mortality rates. Geographic maldistribution of CIT follows patterns similar to other mental health services and professionals: many rural counties in the South and Midwest had no access to CIT in either 2015 or 2020. This may speak to the need to develop rural-specific crisis models, as many models—including the original CIT model and more recent models such as CAHOOTs in Portland Oregon—were developed in an urban context.^1,26^ We found few racial or ethnic disparities by county in access to CIT. This is important, as minoritized racial and ethnic communities are more likely to face poor first responder reactions at baseline, and outcomes including incarceration or arrest-related death are more likely to occur for minoritized racial and ethnic individuals in crisis compared with White people.^17^ However, we did find that counties with access to CIT were more likely to be counties with the most residential segregation. Given the disproportionate impact of police violence on minoritized racial and ethnic communities, future work should examine within-county disparities in access to crisis intervention services, as other work has shown that minoritized racial and ethnic communities tend to be the furthest distance from county health care resources within counties.^27^

We found that Medicaid may help facilitate funding CIT services, with Medicaid eligibility expansions significantly associated with offering CIT services. State Medicaid programs have had several opportunities to develop CIT capacity through Section 1115 waivers, especially IMD payment exclusion waivers for serious mental illness, and the CCBHC demonstration and expansion, which specifically requires participating clinics to offer crisis management services that are available 24 hours a day. To date, more than 400 clinics have received funding through this mechanism. Although adjusted associations were not statistically significant, counties in states that expanded Medicaid or implemented any behavioral health Section 1115 waiver were more likely to have access to CIT in 2020. This is important because under the current fiscal year allocations included in the ARP, state Medicaid agencies now have the statutory authority and resources to invest in crisis intervention services and greater flexibility to develop programs tailored to their communities. States that leverage this new authority may have the opportunity to revitalize the community behavioral health system beyond crisis intervention: through generating a flexible reimbursement structure to empower peers in providing treatment or addressing social determinants of behavioral health through community partnerships. Authorizing 988 provides needed support for behavioral health crises, but local access to crisis care will be required to realize the full potential of these benefits.

### Limitations

This study has limitations, including the possibility that we underestimated CIT availability because a subset of facilities chose not to be listed in the treatment directory, although these facilities would also not be visible to clients seeking services on SAMHSA’s treatment locator. In addition, the N-MHSS does not include police departments in their sample frame, and some police departments offer CIT services. However, CIT programs in police departments tend to be in urban areas, and therefore their inclusion would likely not change the associations that we report in this study. This study is observational; we only report associations.

## Conclusions

In a cross-sectional analysis using directory data from 2015 and 2020, we found that fewer than half of US counties have access to CITs, although most US residents do reside in counties with access to at least 1 CIT. Policies to encourage facilities in rural counties to offer CIT may help realize the potential of the 988 mental health crisis lifeline.

## Notes

Supplement. **eTable 1. **Associations Between Area and State Medicaid Characteristics and Change in County-Level Access to CIT, 2015 to 2020 **eTable 2.** Logistic Regression Results (Main Specification) **eTable 3.** Logistic Regression Results Excluding and Including Measures of Need (Suicide and Overdose Mortality Rates)
