# Supplementary material for: Association of Access to Crisis Intervention Teams With County Sociodemographic Characteristics and State Medicaid Policies and Its Implications for a New Mental Health Crisis Lifeline
Source: JAMA Netw Open. 2022 Jul 15;5(7):e2224803. doi: 10.1001/jamanetworkopen.2022.24803 (PMC9287760; doi:10.1001/jamanetworkopen.2022.24803)
Supplement: Supplement. — eTable 1. Associations Between Area and State Medicaid Characteristics and Change in County-Level Access to CIT, 2015 to 2020 eTable 2. Logistic Regression Results (Main Specification) eTable 3. Logistic Regression Results Excluding and Including Measures of Need (Suicide and Overdose Mortality Rates) [file jamanetwopen-e2224803-s001.pdf]

Supplemental Online Content

Newton H, Beetham T, Busch SH. Association of access to crisis intervention teams with county sociodemographic characteristics and state Medicaid policies and its implications for a new mental health crisis lifeline. *JAMA Netw Open.* 2022;5(7):e2224803.
doi:10.1001/jamanetworkopen.2022.24803

eTable 1. Associations Between Area and State Medicaid Characteristics and Change in County-Level Access to CIT, 2015 to 2020

eTable 2. Logistic Regression Results (Main Specification)

eTable 3. Logistic Regression Results Excluding and Including Measures of Need (Suicide and Overdose Mortality Rates)

This supplemental material has been provided by the authors to give readers additional information about their work.

© 2022 Newton H et al. *JAMA Network Open.*

**eTable 1.** Associations Between Area and State Medicaid Characteristics and Change in County-Level Access to CIT, 2015 to 2020					
	**No access 2015 and 2020**	**Access 2015, No access 2020**	**No access in 2015, Access 2020**	**Access 2015 and 2020**	**p-value**
**N Counties**	1244	268	293	1337	
(% Sample)	(40%)	(9%)	(9%)	(43%)	
County Population in 2020 (total)	28,142,429	12,392,009	14,893,144	274,056,541	
**Area Characteristics**					
**Age Distribution** (1=top quartile)					
Age >55 years	36%	20%	23%	16%	<0.01
**Demographics** (1=top quartile)					
Uninsured	34%	27%	24%	16%	<0.01
Unemployed	23%	25%	22%	28%	0.09
Living at or below FPL	19%	20%	24%	32%	<0.01
**Race/Ethnicity** (1=top quartile)					
White	29%	25%	27%	21%	0.11
Black	24%	23%	25%	26%	0.75
Asian	11%	15%	22%	40%	<0.01
Native Hawaiian/Pacific Islander	20%	24%	23%	30%	0.01
American Indian/Alaskan Native	27%	26%	21%	24%	0.41
Hispanic	23%	21%	23%	28%	0.53
**Residential Segregation** (1=top quartile)	20%	18%	19%	31%	0.02
Most Segregated (Non-White/White)					
**Need for Services**					
**Behavioral health mortality 2020** (mean)	25%	28%	26%	24%	0.98
Drug overdose deaths per 100,000 population	32%	29%	23%	21%	0.00
Suicide deaths per 100,000 population					
**Rurality**	24%	31%	38%	50%	<0.01
Metro (non-rural)	39%	56%	49%	42%	0.08
Micropolitan (rural)	37%	14%	13%	8%	<0.01
Frontier (rural)	36%	20%	23%	16%	<0.01
**State Medicaid Characteristics** (mean percent)					
**Enacted Before 2020:**					
Expanded Medicaid	50%	60%	58%	70%	0.01
Any Behavioral Health Section 1115 Waiver	46%	55%	55%	60%	0.04
IMD Payment Exclusion (substance use disorder)	41%	50%	52%	51%	0.11
IMD Payment Exclusion (mental health)	2%	6%	3%	5%	<0.01
Eligibility Expansions	4%	6%	2%	9%	0.19
Delivery System Reforms	4%	5%	4%	5%	0.78
Community-Based Benefit Expansions	22%	30%	23%	32%	0.09

© 2022 Newton H et al. *JAMA Network Open.*

2015 Certified Community Behavioral Health Clinic Demonstration Planning Grant State	48%	48%	50%	53%	0.53
Per-capita SAMHSA funding related to suicide prevention, crisis intervention, or diversion (1=top-quartile)	22%	25%	18%	26%	0.51
IMD DSH payments (1=top-quartile)	25%	24%	20%	23%	0.79
**Enacted After 2020:**					
American Rescue Plan CIT Development Planning Grant State	27%	32%	31%	39%	0.05
988 Legislation Passed or Pending	35%	32%	42%	42%	0.42

Notes: eTable 1 reproduces Table 1, but instead examines the distribution of area and state characteristics by 4-category change in access to CIT variable (from Figure 1). IMD = Institutions of Mental Disease. DSH = Disproportionate Share Hospital. Three area characteristics have missing observations (measures of drug-related overdose deaths, suicide mortality, and residential segregation), percentages reported in this table reflect the percent among observations with non-missing values (n=1420, 763, 351 respectively).

**eTable 2.** Logistic Regression Results (Main Specification)		
	Dep. Var	
	County-level Access to CIT in 2020 (0,1)	
	Logit	Percentage Point change in likelihood
	Beta<br>(SE)	Point Estimate<br>(SE)
**Area Characteristics**		
**Age Distribution** (1=top quartile)		
Age >55 years	-0.143	-2.961
	(0.167)	(3.507)
**Demographics** (1=top quartile)		
Uninsured	-0.454**	-9.619**
	(0.228)	(4.837)
Median Household Income	-0.116	-2.390
	(0.119)	(2.438)
Unemployed	0.104	2.142
	(0.131)	(2.697)
**Race/Ethnicity** (1=top quartile)		
White	0.103	2.121
	(0.170)	(3.487)
Black	-0.167	-3.451
	(0.221)	(4.584)
Hispanic	0.179	3.679
	(0.197)	(4.005)
AI/AN	-0.0332	-0.683
	(0.204)	(4.200)
Asian	1.202***	24.848***
	(0.117)	(2.183)
NH/PI	-0.0359	-0.738
	(0.126)	(3.160)
**Residential Segregation** (1=top quartile)		
Most Segregated (Non-White/White)	0.386**	7.928**
	(0.158)	(3.209)
**Rurality**		
Metro (urban)	*Reference*	*Reference*
Micropolitan (rural)	-0.237***	-5.037***
	(0.0911)	(1.980)
Frontier (rural)	-1.187***	-25.453***
	(0.182)	(3.725)
**Need for Services**		
**Behavioral Health Mortality (2020)** (1=top-quartile)		
Suicide deaths per 100,000 population	-	-
	-	-
Drug overdose deaths per 100,000 population	-	-
	-	-

© 2022 Newton H et al. *JAMA Network Open.*

**State Medicaid Characteristics**		
**Enacted Before 2020:**		
Expanded Medicaid	0.476*	10.037*
	(0.264)	(5.604)
Any Behavioral Health Section 1115 Waiver	0.824*	17.163
	(0.453)	(9.221)
IMD Payment Exclusion (substance use disorder)	-0.914*	-17.483*
	(0.491)	(8.399)
IMD Payment Exclusion (mental health)	0.367	7.545
	(0.555)	(11.495)
Community-Based Benefit Expansions	-0.0591	-1.215
	(0.294)	(6.050)
Eligibility Expansions	0.0601	1.236
	(0.462)	(9.507)
Delivery System Reforms	-0.419	-8.688
	(0.335)	(6.956)
2015 Certified Community Behavioral Health Clinic Demonstration Planning Grant State	-0.0270	-0.556
	(0.383)	(7.872)
Per-capita SAMHSA funding related to suicide prevention, crisis intervention, or diversion (1=top-quartile)	-0.354	-7.290
	(0.292)	(5.994)
IMD DSH payments (1=top-quartile)	-0.167	-3.442
	(0.297)	(6.191)
**Enacted After 2020:**		
American Rescue Plan CIT Development Planning Grant State	0.425*	8.752
	(0.225)	(4.558)
988 Legislation Passed or Pending	0.141	2.907
	(0.212)	(4.351)
Constant	-0.0425	
	(0.321)	
**Observations**	2,790	2,790
*** p<0.01, ** p<0.05, * p<0.1		

© 2022 Newton H et al. *JAMA Network Open.*

**eTable 3.** Logistic Regression Results Excluding and Including Measures of Need (Suicide and Overdose Mortality Rates)		
	Dep. Var	
	County-level Access to CIT in 2020 (0,1)	
	Excluding measures of Need (results shown in manuscript)	Including Measures of Need (results not shown in manuscript)
	Beta<br>(SE)	Beta<br>(SE)
**Area Characteristics**		
**Age Distribution** (1=top quartile)		
Age >55 years	-0.143	0.271
	(0.167)	(0.227)
**Demographics** (1=top quartile)		
Uninsured	-0.454**	-0.245
	(0.228)	(0.215)
Median Household Income	-0.116	-0.311*
	(0.119)	(0.180)
Unemployed	0.104	-0.0348
	(0.131)	(0.160)
**Race/Ethnicity** (1=top quartile)		
White	0.103	-0.119
	(0.170)	(0.153)
Black	-0.167	-0.0380
	(0.221)	(0.196)
Hispanic	0.179	0.183
	(0.197)	(0.237)
American Indian/Alaskan Native	-0.0332	0.176
	(0.204)	(0.244)
Asian	1.202***	1.155***
	(0.117)	(0.132)
Native Hawaiian/Pacific Islander	-0.0359	-0.298*
	(0.126)	(0.160)
**Residential Segregation** (1=top quartile)		
Most Segregated (Non-White/White)	0.386**	0.536***
	(0.158)	(0.169)
**Rurality**		
Metro (urban)	*Reference*	*Reference*
Micropolitan (rural)	-0.237***	-0.381***
	(0.0911)	(0.144)
Frontier (rural)	-1.187***	-0.694*
	(0.182)	(0.398)
**Need for Services**		
**Behavioral Health Mortality (2020)** (1=top-quartile)		
Suicide deaths per 100,000 population	-	-0.0320

© 2022 Newton H et al. *JAMA Network Open.*

	-	(0.184)
Drug overdose deaths per 100,000 population	-	-0.224
	-	(0.153)
**State Medicaid Characteristics**		
**Enacted Before 2020:**		
Expanded Medicaid	0.476*	0.872***
	(0.264)	(0.218)
Any Behavioral Health Section 1115 Waiver	0.824*	0.764**
	(0.453)	(0.385)
IMD Payment Exclusion (substance use disorder)	-0.914*	-0.948**
	(0.491)	(0.479)
IMD Payment Exclusion (mental health)	0.367	0.184
	(0.555)	(0.531)
Community-Based Benefit Expansions	-0.0591	-0.148
	(0.294)	(0.213)
Eligibility Expansions	0.0601	0.268
	(0.462)	(0.456)
Delivery System Reforms	-0.419	-0.452
	(0.335)	(0.425)
2015 Certified Community Behavioral Health Clinic Demonstration Planning Grant State	-0.0270	-0.206
	(0.383)	(0.302)
Per-capita SAMHSA funding related to suicide prevention, crisis intervention, or diversion (1=top-quartile)	-0.354	-0.274
	(0.292)	(0.292)
IMD DSH payments (1=top-quartile)	-0.167	-0.220
	(0.297)	(0.250)
**Enacted After 2020:**		
American Rescue Plan CIT Development Planning Grant State	0.425*	0.362*
	(0.225)	(0.194)
988 Legislation Passed or Pending	0.141	0.359
	(0.212)	(0.240)
Constant	-0.0425	0.268
	(0.321)	(0.378)
**Observations**	2,790	1,692
*** p<0.01, ** p<0.05, * p<0.1		

© 2022 Newton H et al. *JAMA Network Open.*
